# Mitochondrial Junction Region as Genotyping Marker for *Cyclospora cayetanensis*

**DOI:** 10.3201/eid2507.181447

**Published:** 2019-07

**Authors:** Fernanda S. Nascimento, John R. Barta, Julia Whale, Jessica N. Hofstetter, Shannon Casillas, Joel Barratt, Eldin Talundzic, Michael J. Arrowood, Yvonne Qvarnstrom

**Affiliations:** Centers for Disease Control and Prevention, Atlanta, Georgia, USA (F.S. Nascimento, J.N. Hofstetter, S. Casillas, J. Barratt, E. Talundzic, M.J. Arrowood, Y. Qvarnstrom);; University of Guelph, Guelph, Ontario, Canada (J.R. Barta, J. Whale)

**Keywords:** Cyclospora cayetanensis, cyclosporiasis, parasites, genotyping, mitochondrial DNA, mitochondrial junction region, genotyping marker, water-borne infections, food-borne infections, food safety, epidemiology, enteric infections, United States

## Abstract

Cyclosporiasis is an infection caused by *Cyclospora cayetanensis*, which is acquired by consumption of contaminated fresh food or water. In the United States, cases of cyclosporiasis are often associated with foodborne outbreaks linked to imported fresh produce or travel to disease-endemic countries. Epidemiologic investigation has been the primary method for linking outbreak cases. A molecular typing marker that can identify genetically related samples would be helpful in tracking outbreaks. We evaluated the mitochondrial junction region as a potential genotyping marker. We tested stool samples from 134 laboratory-confirmed cases in the United States by using PCR and Sanger sequencing. All but 2 samples were successfully typed and divided into 14 sequence types. Typing results were identical among samples within each epidemiologically defined case cluster for 7 of 10 clusters. These findings suggest that this marker can distinguish between distinct case clusters and might be helpful during cyclosporiasis outbreak investigations.

*Cyclospora cayetanensis* is a coccidian parasite that causes human cyclosporiasis, an enteric infection associated with consumption of fecally contaminated fresh food or water harboring sporulated oocysts of this parasite. Cyclosporiasis most commonly occurs in tropical and subtropical regions (*1*). Cases in temperate regions are often associated with travel to countries where the disease is endemic or with foodborne outbreaks linked to various types of imported fresh produce (*2*–*4*). Cases in Canada and the United Kingdom have in recent years been increasingly associated with travel to the Riviera Maya and Cancun areas in Mexico (*5*,*6*).

In 2017, the Centers for Disease Control and Prevention was notified of 1,065 laboratory-confirmed cases of cyclosporiasis in the United States, of which >56% were domestically acquired (*7*). A case–control study identified green onions as being strongly associated with cyclosporiasis cases among 16 persons who dined at a Mediterranean-style restaurant chain in the Houston, Texas, area in 2017 (*8*). However, despite extensive epidemiologic investigation and trace-back efforts, the specific exposures associated with most of the cases in 2017 were not identified. The time lag between exposure to the contaminated source, the onset of clinical symptoms, and the epidemiologic investigation can be several weeks. Consequently, case-patients might be asked to recall relevant food exposure weeks to months before the interview and may not recall specific food exposures or identify ingredients included in certain dishes.

A validated molecular typing marker could help to improve our understanding of cyclosporiasis epidemiology and facilitate identification and investigation of disease clusters. Recent advances in next-generation sequencing have enabled whole-genome sequencing of the *C. cayetanensis* parasite (*9*,*10*), including its organellar genomes derived from the apicoplast (*11*,*12*) and mitochondrion (*12*–*14*). These advances facilitated development of a multilocus sequence typing (MLST) method based on 5 microsatellites. However, when this method was applied to stool samples, data were successfully obtained for all 5 loci for <60% of samples (*15*,*16*). In addition, the epidemiologic usefulness of the MLST method in outbreak investigations is currently unknown.

*C. cayetanensis* is a member of the phylum Apicomplexa. Its mitochondrial genome is ≈6.3 kb and is a linear molecule with >2 copies arranged in a concatemeric structure with a head-tail configuration (*12*–*14*). Comparison of the mitochondrial genomes of *C. cayetanensis* isolates from the United States and China showed only minor sequence variations (*12*). However, mitochondrial genomes from different isolates vary in length and seem to have a greater amount of variation in the junction area between the genome copies (*17*). The purpose of this study was to explore the sequence variation of this junction area of the mitochondrial genome and evaluate it as a potential typing marker for linking cyclosporiasis cases.

## Methods

### Sample Collection

Stool samples from 134 patients given a diagnosis of cyclosporiasis during 2013–2016 were sent to the Centers for Disease Control and Prevention from state public health laboratories in the United States for confirmatory diagnostic testing or as part of a research study. The samples had been collected in PCR-friendly stool preservatives (e.g., Zn-PVA) or transport medium (e.g., Cary-Blair) and were confirmed positive for *Cyclospora* sp. parasites by ultraviolet fluorescence microscopy (*18*). The samples were collected in the following states and years: Florida (n = 1), Iowa (n = 7), and Texas (n = 6), 2013; Maine (n = 4), Massachusetts (n = 5), Michigan (n = 6), Ohio (n = 1), Pennsylvania (n = 2), South Carolina (n = 3), and Texas (n = 24), 2014; Georgia (n = 1), Illinois (n = 1), Texas (n = 42) and Wisconsin (n = 6), 2015; and Florida (n = 4), Georgia (n = 1), Nebraska (n = 7), and Texas (n = 13), 2016.

### Epidemiologic Investigations and Classification

We defined an outbreak as >2 epidemiologically linked cases (e.g., a cluster of cases in persons linked to a restaurant, grocery store, or social event). We defined a temporospatial cluster as cases that occurred in the same geographic area (e.g., in the same community or town) and had illness onset dates around the same time (e.g., within ≈15 days of each other). Epidemiologic evidence for linking cases with common exposures (e.g., restaurant, grocery store, or social events) is typically stronger than for temporospatial clusters. We defined an international travel–associated case as a case in a person who spent >1 day during their pertinent incubation period (i.e., 14 days before illness onset) outside the United States.

### DNA Extraction and Molecular Detection

We washed 2 mL of each stool twice with phosphate-buffered saline, pH 7.4, and used 500 µL of the feces for DNA extraction by using the UNEX method, as described elsewhere (*19*). We amplified the mitochondrial junction region in a 25-μL PCR by using the NEBNext Q5 Hot Start HiFi PCR Master Mix (New England Biolabs, https://www.neb.com), 400 nmol/L of each of the forward (cyclo_mit-100F: TACCAAAGCATCCATCTACAGC) and reverse (cyclo_mit-54R: CCCAAGCAATCGGATCGTGTT) primers, and 1 μL of the DNA sample. The cycling conditions were 98°C for 2 min, followed by 35 cycles of 98°C for 15 s, 66°C for 15 s, and 72°C for 30 s, and a final extension at 72°C for 5 min. PCR products of ≈200 bp were visualized by electrophoresis on a 1.5% agarose gel stained with ethidium bromide. We purified the PCR products by using the Monarch PCR and DNA Cleanup Kit (New England Biolabs) and sequenced them on an ABI PRISM 3130xl Genetic Analyzer (Applied Biosystems, https://www.thermofisher.com) in both directions by using the PCR primers and BigDye Terminator V3.1 chemistry (Applied Biosystems). We used the DyeEx 2.0 Spin Kit (QIAGEN, https://www.qiagen.com) to remove unincorporated dyes.

### Data Analysis and Sequences

We aligned forward and reverse sequence reads by using the MAFFT version 7.222 (*20*) plug-in in Geneious R11 (*21*). The variant types of the mitochondrial junction are available in GenBank (accession nos. MH430075–88).

### Ethics

We used stool samples in accordance with the Human Subjects Research Protocol (use of coded specimens for *Cyclospora* genomics research). This protocol was approved by the Human Research Protection Office in the Center for Global Health, Centers for Disease Control and Prevention (#2014–107).

## Results

We amplified the mitochondrial junction region from 133 (99%) of 134 samples from patients with confirmed diagnosis of cyclosporiasis; 1 sample from Iowa did not show any visible band after amplification. Sanger sequencing from 132 of these samples generated data of sufficient quality for analysis in both forward and reverse direction; 1 sample from Michigan did not produce readable sequences. The mitochondrial junction region of *C. cayetanensis* exhibited a high degree of variability between samples because of 3 variations of a 15-nt motif referred to as type I, TAGTATTATTT**A**TAA; type II, TAGTATTATTT**T**TAA; and type III, TAGTATTATTT**TA**AA (variant nucleotides are shown in bold) (Appendix Figure). These repeats were present in 2–5 copies in various combinations and resulted in different lengths and composition of the mitochondrial junction. On the basis of the number of repeats, we divided sequences into 4 main groups designated Cmt154, Cmt169, Cmt184, and Cmt199. Each main group could be further divided into 2–5 sequence types on the basis of the repeat motifs and 3 single-nucleotide polymorphisms (SNPs) present downstream of the repeat region. The sequence types were designated with an arbitrary letter following the group number (e.g., Cmt154.A, Cmt154.B). The combination of repeat motifs and SNPs resulted in 14 unique mitochondrial junction sequence (Cmt) types among the 132 samples analyzed (Table 1).

**Table 1:** *Cyclospora cayetanensis* mitochondrial junction types identified among 132 samples collected in different states, United States, 2013–2016*

Mitochondrial junction type	No. samples	Collection year (state)
Cmt154.A	50	2013 (TX); 2014 (MI, SC, TX); 2015 (GA, IL, TX, WI); 2016 (FL, GA, NE, TX)
Cmt154.B	34	2014–2016 (TX); 2016 (NE)
Cmt154.C	2	2013 (TX); 2015 (TX)
Cmt154.D	1	2015 (TX)
Cmt169.A	12	2013 (FL, TX); 2014 (MA, OH, PA)
Cmt169.B	7	2014–2016 (TX); 2015 (WI)
Cmt184.A	6	2013 (IA)
Cmt184.B	7	2014 (MA, MI, PA, TX); 2016 (FL)
Cmt184.C	5	2014 (ME); 2015 (TX)
Cmt184.D	3	2014 (MI, TX); 2016 (NE)
Cmt184.E	1	2013 (TX)
Cmt199.A	2	2014 (TX), 2016 (NE)
Cmt199.B	1	2014 (MA)
Cmt199.C	1	2016 (FL)

*Cmt, *Cyclospora* mitochondrial junction.

We determined the relationship between different Cmt sequences and their distribution among samples analyzed from epidemiologically linked or sporadic cases (Figure). This information includes all Cmt types publicly available in GenBank as of August 2018, including type Cmt214.A, which is the longest type described so far but was not encountered in this study. The Cmt types have 2–6 copies of the 3 different 15-mer repeats in various combinations. The predominant type, Cmt154.A, was found in 50 samples in this study, including 16 case-patients with a travel history to Mexico, 1 case-patient with a travel history to Costa Rica, and 14 case-patients linked to outbreaks/clusters in South Carolina (2014), Texas (2015–2016), and Wisconsin (2015). A total of 34 samples typed as Cmt154.B, including 11 samples from patients with a travel history to Mexico, 9 cases linked to several restaurant-associated outbreaks in Texas (2015), and 1 case linked to an event-associated outbreak in Michigan (2014). We also provide detailed typing and epidemiologic information for all 132 samples (Appendix Table).

**Figure:**
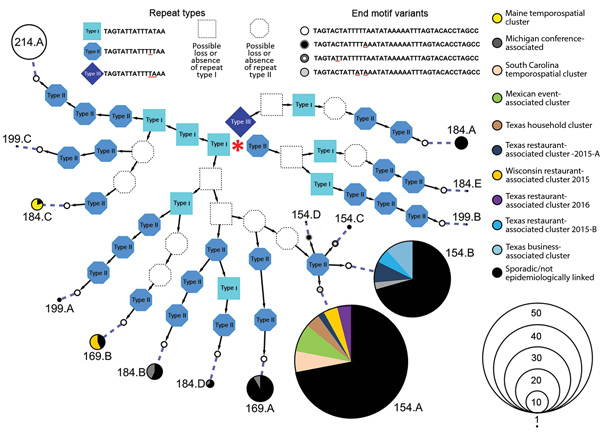
Relationships between detected *Cyclospora* mitochondrial junction (Cmt) types, United States. Fourteen unique Cmt types were detected. Cmt214.A (top left) was not detected in this study but was reported previously (GenBank accession no. MH430089.1); it represents the type with the largest number of 15-mer repeats (total 6) and is therefore included as reference for comparison. Three different 15-mer repeat sequences are known, and each Cmt type possesses 2–6 of these 15-mer repeats in various combinations. The sequence of each mt junction type can be elucidated from this figure starting with the first repeat, indicated by the red central asterisk, and then following the arrows to the end motif. A dashed line links the sequence to a pie chart that provides epidemiologic information. The size of the pie chart represents the number of times this particular Cmt type was detected. For instance, type 154.A was detected in 50 samples (as reflected by the scale) and represents the most common type. Red underlined letters indicate variable sites that exist in the end motif and 15-mer repeats.

A total of 37 of the analyzed samples were epidemiologically associated with 10 outbreaks or temporospatial case clusters (Table 2). Seven of these clusters had identical typing results among the samples within each cluster: 2 temporospatial clusters in South Carolina and Maine in 2014, an event in Mexico in 2015, a Texas household in 2015, and 3 restaurant outbreaks in Texas (2 in 2015 and 1 in 2016). Conversely, 2 restaurant-associated outbreaks in Wisconsin and Texas in 2015, and an event-associated outbreak in Michigan in 2014 had >2 types identified within each cluster.

**Table 2:** Distribution of *Cyclospora cayetanensis* mitochondrial junction types detected in epidemiologically linked samples, United States*

Collection state and year	Epidemiologic known link to case cluster/outbreak	Sample no.	International travel within 2 weeks before symptom onset	Cmt type
Maine 2014	Maine temporospatial cluster†	HCME548_14	No	Cmt184.C
		HCME550_14	No	Cmt184.C
		HCME552_14	No	Cmt184.C
		HCME298_14	No	Cmt184.C
Michigan 2014	Michigan event-associated cluster	HCMI030_14	Unknown	Cmt154.B
		HCMI040_14	No	Cmt184.D
		HCMI029_14	No	Cmt184.B
		HCMI039_14	Unknown	Cmt184.B
Pennsylvania 2014		HCPA556_14	No	Cmt184.B
		HCPA962_14	Unknown	Cmt169.A
South Carolina 2014	South Carolina temporospatial cluster†	HCSC052_14	No	Cmt154.A
		HCSC053_14	No	Cmt154.A
		HCSC054_14	No	Cmt154.A
Texas 2015	Mexican event-associated cluster	HCTX208_15	Mexico/Tulum	Cmt154.A
		HCTX219_15	Mexico/Tulum	Cmt154.A
		HCTX220_15	Mexico/Tulum	Cmt154.A
		HCTX547_15	Mexico/Tulum	Cmt154.A
	Texas household cluster‡	HCTX354_15	Mexico/Riviera Maya	Cmt154.A
		HCTX355_15	Mexico/Riviera Maya	Cmt154.A
	Texas restaurant-associated cluster 2015-A	HCTX353_15	No	Cmt154.A
		HCTX540_15	No	Cmt154.B
		HCTX551_15	No	Cmt154.B
		HCTX555_15	No	Cmt154.B
	Texas restaurant-associated cluster 2015-B	HCTX356_15	No	Cmt154.B
		HCTX357_15	No	Cmt154.B
	Texas local business-associated cluster	HCTX204_15	Mexico/Cozumel	Cmt154.B
		HCTX205_15	No	Cmt154.B
		HCTX206_15	No	Cmt154.B
		HCTX538_15	No	Cmt154.B
Wisconsin 2015	Wisconsin restaurant-associated cluster 2015	HCWI001_15	No	Cmt154.A
		HCWI003_15	No	Cmt154.A
		HCWI002_15	No	Cmt169.B
		HCWI004_15	No	Cmt169.B
		HCWI005_15	No	Cmt169.B
		HCWI006_15	No	Cmt169.B
Texas 2016	Texas restaurant-associated cluster 2016	HCTX471_16	No	Cmt154.A
		HCTX474_16	No	Cmt154.A

*Cmt, *Cyclospora* mitochondrial junction.

†The terminology temporospatial cluster is used here for cases that were not linked to a particular establishment or event but were temporally and geographically clustered.

‡Case-patients were a married couple who traveled together to Riviera Maya, Mexico, during their incubation period. Because they did not spend the entire 14-d incubation period in Mexico, it is unclear whether they became infected in Texas or Mexico.

## Discussion

We investigated DNA sequence variations in the short junction segment of the mitochondrial genome in *C. cayetanensis* parasites. We distinguished 14 Cmt types among 132 samples collected in the United States during 2013–2016 on the basis of sequence length and the SNPs in this region. The variability of the mitochondrial junction region detected in our study adds to the current knowledge of the structure of the *C. cayetanensis* mitochondrial genome. A recently published strategy for assembly and comparison of mitochondrial genomes of *C. cayetanensis* reported a variable number of 15-mer repeats in the terminal region of the mitochondrial genome (*17*), a finding that we confirmed and expanded upon in our study. The sequence of type Cmt169.B, which was found in 6 samples in our study, is identical to the mitochondrial junction sequence found in a previously reported sample from Nepal (GenBank accession no. KP231180.1) (*14*). The most distinct mitochondrial genome reported so far is from an isolate from China (*12*), which, on the basis of the draft genome, has only 1 copy of the 15-mer repeat.

The copy number of the mitochondrial genome is still unknown for *C*. *cayetanensis*. Tang et al. (*12*) estimated 513 copies of the mitochondrial genome for each nuclear genome on the basis of the relative proportion of whole-genome sequencing reads mapped to each genome. However, this estimate seems high compared with the mitochondrial copy number in other apicomplexan parasites (e.g., 50 copies/nuclear genome in *Eimeria tenella* [*22*], 20 copies/nuclear genome in *Plasmodium falciparum* [*23*], and 150 copies/nuclear genome in *P. yoelli* [*24*]). Nevertheless, targeting a high copy number locus provides the greatest opportunity for successful amplification directly from clinical samples. We successfully amplified and sequenced the mitochondrial junction in 98.5% of the samples in this study. In contrast, an MLST method based on 5 microsatellite loci in the *C. cayetanensis* nuclear genome resulted in interpretable data from only 53%–59% of samples tested (*15*,*16*).

This study included >2 samples from 10 outbreaks associated with restaurants, specific events, or temporospatial case clusters. Samples from 7 of these clusters/outbreaks had identical typing results for all linked cases, and 3 clusters/outbreaks had linked cases that typed differently. Instances in which the same cluster showed >1 distinct type included an outbreak in Michigan (2014) in which 4 types were detected among 6 patients, an outbreak in Texas (2015) in which 1 patient had a type distinct from the other 3 patients, and an outbreak in Wisconsin (2015) in which 2 different types were detected among 6 patients. As suggested by Guo et al. (*15*), the presence of >1 type in a cluster might be indicative of produce contaminated with mixed populations of *C. cayetanensis*.

To date, epidemiologic investigations of cyclosporiasis cases and outbreaks have been limited by the lack of molecular typing methods that can reliably differentiate isolates of *C. cayetanensis. *Our study suggests that PCR amplification and DNA sequencing of a short region of the mitochondrial genome might provide useful typing information to aid such investigations. Performing amplicon deep sequencing of the Cmt region by using next-generation sequencing methods might also enable analysis of clinical or environmental samples containing multiple genotypes. Although further studies are required, including sampling from broader geographic areas, we propose that the mitochondrial junction region of *C. cayetanensis* shows promise as a molecular typing marker for this human pathogen.

Appendix Additional information on mitochondrial junction region as genotyping marker for *Cyclospora cayetanensis*.
