# Supplementary material for: Mitochondrial Junction Region as Genotyping Marker for Cyclospora cayetanensis
Source: Emerg Infect Dis. 2019 Jul;25(7):1314–9. doi: 10.3201/eid2507.181447 (PMC6590752; doi:10.3201/eid2507.181447)
Supplement: Appendix — Additional information on mitochondrial junction region as genotyping marker for Cyclospora cayetanensis. [file 18-1447-Techapp-s1.pdf]

Article DOI: https://doi.org/10.3201/eid2507.181447

Mitochondrial Junction Region as Genotyping Marker for *Cyclospora cayetanensis*

Appendix

Appendix Table. Distribution of 14 types of *Cyclospora cayetanensis* found among 132 samples analyzed, including available epidemiologic information about linkage to case clusters and international travel, United States

Cmt type*	Sample no.	Collection state and year	Epidemiologic known link to case cluster/outbreak	International travel within 2 weeks before symptom onset
Cmt154.A	HCTX119_13	Texas 2013	Unknown	Unknown
	HCTX120_13		Unknown	Unknown
	HCTX363_13		Unknown	Unknown
	HCMI148_14	Michigan 2014	Unknown	Unknown
	HCSC052_14	South Carolina 2014	South Carolina	No
	HCSC053_14		temporospatial† cluster	No
	HCSC054_14			No
	HCTX541_14	Texas 2014	No	No
	HCTX543_14		No‡	No
	HCTX550_14		No	No
	HCTX553_14		No	No
	HCTX556_14		No	No
	HCTX558_14		No	No
	HCTX564_14		No	No
	HCTX566_14		No	No
	HCTX772_14		No	No
	HCTX693_14		No	No
	HCTX681_14		No	No
	HCGA001_15	Georgia 2015	No‡	No
	HCIL001_15	Illinois 2015	Unknown	Unknown
	HCTX208_15	Texas 2015	Mexican event-associated	Mexico/Tulum
	HCTX219_15		cluster	Mexico/Tulum
	HCTX220_15			Mexico/Tulum
	HCTX547_15			Mexico/Tulum
	HCTX227_15	Texas 2015	No	Mexico/Playa del Carmen
	HCTX354_15	Texas 2015	Texas household cluster§	Mexico/Riviera Maya
	HCTX355_15			Mexico/Riviera Maya
	HCTX236_15	Texas 2015	No	Mexico/Cancun
	HCTX544_15		No	Mexico/Tulum
	HCTX550_15		No	Mexico
	HCTX223_15		No	Costa Rica
	HCTX520_15		No	No
	HCTX543_15		No	No
	HCTX518_15		No‡	No
	HCTX451_15		No	Mexico/Cancun
	HCTX353_15	Texas 2015	Texas restaurant-associated cluster 2015-A	No
	HCWI001_15	Wisconsin 2015	Wisconsin restaurant-	No
	HCWI003_15		associated cluster 2015	No
	HCFL006_16	Florida 2016	No	No
	HCGA001_16	Georgia 2016	Unknown	Unknown
	HCNE181_16	Nebraska 2016	No	No
	HCNE184_16		No	No
	HCTX471_16	Texas 2016	Texas restaurant-associated	No
	HCTX474_16		cluster 2016	No
	HCTX462_16	Texas 2016	No	Mexico
	HCTX467_16		No	Mexico/Jalisco
	HCTX475_16		No	Mexico/Cancun
	HCTX493_16		No	Mexico
	HCTX494_16		No	Mexico/Veracruz
	HCTX500_16		No	No
Cmt154.B	HCMI030_14	Michigan 2014	Michigan conference-associated cluster	Unknown
	HCTX565_14	Texas 2014	No	Unknown
	HCTX538_14		No	Mexico/Cancun
	HCTX545_14		No	No

Cmt type*	Sample no.	Collection state and year	Epidemiologic known link to case cluster/outbreak	International travel within 2 weeks before symptom onset
	HCTX548_14		No	No
	HCTX559_14		No	No
	HCTX560_14		No	Mexico/Puerto Vallarta
	HCTX567_14		No	No
	HCTX697_14		No	Mexico
	HCTX540_15	Texas 2015	Texas restaurant-associated cluster 2015-A	No
	HCTX551_15			No
	HCTX555_15			No
	HCTX356_15	Texas 2015	Texas restaurant-associated cluster 2015-B	No
	HCTX357_15			No
	HCTX204_15	Texas 2015	Texas business-associated cluster	Mexico/Cozumel
	HCTX205_15			No
	HCTX206_15			No
	HCTX538_15			No
	HCTX522_15	Texas 2015	No‡	No
	HCTX524_15		No‡	No
	HCTX556_15		No‡	No
	HCTX230_15		No	Mexico/Riviera Maya
	HCTX554_15		No	Mexico/Mexico City
	HCTX531_15		No	Mexico
	HCTX229_15		No	No
	HCTX539_15		No	No
	HCTX523_15		No	No
	HCTX526_15		No	No
	HCNE185_16	Nebraska 2016	No	No
	HCNE186_16		No	Mexico/Playa del Carmen
	HCTX503_16	Texas 2016	No	Mexico/Cancun
	HCTX504_16		No	Mexico/Playa del Carmen
	HCTX468_16		No	Mexico/Cancun
	HCTX476_16		No	Mexico/Cancun
Cmt154.C	HCTX141_13	Texas 2013	Unknown	Unknown
	HCTX553_15	Texas 2015	No	No
Cmt154.D	HCTX234_15		No	Mexico/Puerto Morales
Cmt169.A	HCFL747_13	Florida 2013	Unknown	Unknown
	HCTX135_13	Texas 2013	Unknown	Unknown
	HCMA003_14	Massachusetts 2014	No	No
	HCMA011_14	Massachusetts 2014	No	Unknown
	HCPA962_14	Pennsylvania 2014	Michigan conference-associated cluster	Unknown
	HCOH722_14	Ohio 2014	Unknown	Unknown
	HCTX571_14	Texas 2014	No	No
	HCTX199_15	Texas 2015	No	Mexico/Playa del Carmen
	HCTX221_15		No	Unknown
	HCTX545_15		No	Mexico/Playa del Carmen
	HCFL007_16	Florida 2016	No	Mexico/Cancun
	HCNE180_16	Nebraska 2016	No	No
Cmt169.B	HCTX563_14	Texas 2014	No	No
	HCTX213_15	Texas 2015	No	Unknown
	HCWI002_15	Wisconsin 2015	Wisconsin restaurant-associated cluster 2015	No
	HCWI004_15			No
	HCWI005_15			No
	HCWI006_15			No
	HCTX495_16	Texas 2016	No	El Salvador
Cmt184.A	HCIA001_13	Iowa 2013	Unknown	Unknown
	HCIA002_13		Unknown	Unknown
	HCIA003_13		Unknown	Unknown
	HCIA004_13		Unknown	Unknown
	HCIA005_13		Unknown	Unknown
	HCIA006_13		Unknown	Unknown
Cmt184.B	HCMA005_14	Massachusetts 2014	No	No
	HCMA007_14		No	No
	HCMI029_14	Michigan 2014	Michigan conference-associated cluster	No
	HCMI039_14			Unknown
	HCPA556_14	Pennsylvania 2014		No
	HCTX592_14	Texas 2014	No‡	No
	HCFL005_16	Florida 2016	No	No
Cmt184.C	HCME548_14	Maine 2014	Maine temporospatial§ cluster	No
	HCME550_14			No
	HCME552_14			No
	HCME298_14			No
	HCTX215_15	Texas 2015	No	Mexico

Cmt type*	Sample no.	Collection state and year	Epidemiologic known link to case cluster/outbreak	International travel within 2 weeks before symptom onset
Cmt184.D	HCMI040_14	Michigan 2014	Michigan conference-associated cluster	No
	HCTX535_14	Texas 2014	No	No
	HCNE182_16	Nebraska 2016	Unknown	Unknown
Cmt184.E	HCTX145_13	Texas 2013	Unknown	Unknown
Cmt199.A	HCTX536_14	Texas 2014	No	No
	HCNE183_16	Nebraska 2016	No	Mexico/Cancun
Cmt199.B	HCMA001_14	Massachusetts 2014	No	No
Cmt199.C	HCFL003_16	Florida 2016	No	No

*Cmt, *Cyclospora* mitochondrial junction.
†The terminology temporospatial cluster is used here for cases that were not linked to a particular establishment or event but were temporally and geographically clustered.
‡Epidemiologically linked samples but only 1 sample tested per cluster.
§Case-patients were a married couple who traveled together to Riviera Maya, Mexico, during their incubation period. Because they did not spend the entire 14-d incubation period in Mexico, it is unclear whether they became infected in Texas or Mexico.

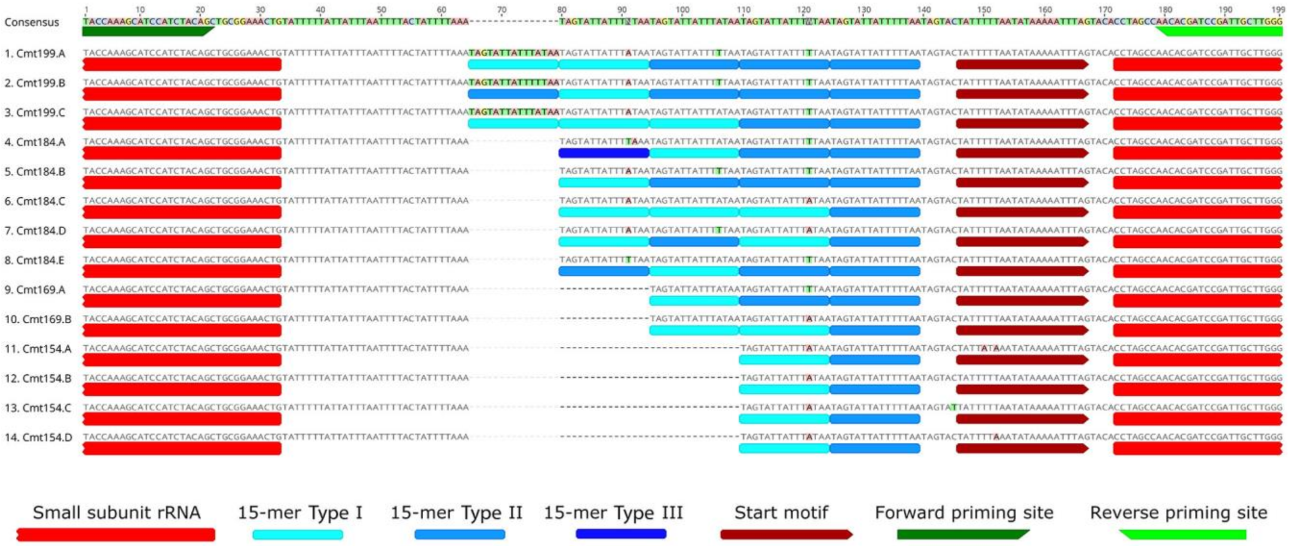

Appendix Figure. Alignment of 14 *Cyclospora* mitochondrial junction (Cmt) types identified from 132 samples analyzed, United States. The 3 different 15-mer motifs are annotated and the single-nucleotide polymorphisms within and outside the repeats are highlighted, indicating all variation among types. Other annotations include primer binding areas, a portion of the mitochondrial small subunit rRNA, and a start motif. The alignment and figure were generated by using Geneious 11.0.3 (https://www.geneious.com). Dashes indicate gaps (absence of nucleotides).
